# Leveraging Neural ODEs for Population Pharmacokinetics of Dalbavancin in Sparse Clinical Data

**DOI:** 10.3390/e27060602

**Published:** 2025-06-05

**Authors:** Tommaso Giacometti, Ettore Rocchi, Pier Giorgio Cojutti, Federico Magnani, Daniel Remondini, Federico Pea, Gastone Castellani

**Affiliations:** 1 Department of Physics and Astronomy, Alma Mater Studiorum, University of Bologna, 40126 Bologna, Italy; tommaso.giacometti5@unibo.it (T.G.); daniel.remondini@unibo.it (D.R.); 2 INFN Istituto Nazionale di Fisica Nucleare, 40127 Bologna, Italy; 3 Department of Medical and Surgical Sciences, Alma Mater Studiorum, University of Bologna, 40126 Bologna, Italy; ettore.rocchi3@unibo.it (E.R.); piergiorgio.cojutti@unibo.it (P.G.C.); federico.magnani9@unibo.it (F.M.); federico.pea@unibo.it (F.P.); 4 IRCCS Azienda Ospedaliero-Universitaria di Bologna, 40128 Bologna, Italy; 5 Clinical Pharmacology Unit, Department for Integrated Infectious Risk Management, IRCCS Azienda Ospedaliero-Universitaria di Bologna, 40128 Bologna, Italy

**Keywords:** neural ODE, deep learning, population pharmacokinetics, precision medicine

## Abstract

This study investigates the use of Neural Ordinary Differential Equations (NODEs) as an alternative to traditional compartmental models and Nonlinear Mixed-Effects (NLME) models for drug concentration prediction in pharmacokinetics. Unlike standard models that rely on strong assumptions and often struggle with high-dimensional covariate relationships, NODEs offer a data-driven approach, learning differential equations directly from data while integrating covariates. To evaluate their performance, NODEs were applied to a real-world Dalbavancin pharmacokinetic dataset comprising 218 patients and compared against a two-compartment model and an NLME within a cross-validation framework, which ensures an evaluation of robustness. Given the challenge of limited data availability, a data augmentation strategy was employed to pre-train NODEs. Their predictive performance was assessed both with and without covariates, while model explainability was analyzed using Shapley additive explanations (SHAP) values. Results show that, in the absence of covariates, NODEs performed comparably to state-of-the-art NLME models. However, when covariates were incorporated, NODEs demonstrated superior predictive accuracy. SHAP analyses further revealed how NODEs leverage covariates in their predictions. These results establish NODEs as a promising alternative for pharmacokinetic modeling, particularly in capturing complex covariate interactions, even when dealing with sparse and small datasets, thus paving the way for improved drug concentration predictions and personalized treatment strategies in precision medicine.

## 1. Introduction

Recent advances in precision medicine, coupled with emerging models and breakthroughs in machine learning (ML), have underscored the growing need for innovation in medical research and practice [1]. Compartment models, in which the human body is represented as a system of interconnected compartments that exchange materials at defined rates, are among the most widely used models for fitting pharmacokinetic data [2]. Furthermore, these models can be estimated using Nonlinear Mixed Effects (NLME) modeling [3,4], a widely adopted approach in population pharmacokinetics. NLME models incorporate two types of parameters: fixed effects, which are constant across the population and capture relationships with observed covariates (e.g., dose, time, body weight); and random effects, which account for inter-individual variability. The random effects are typically assumed to follow a multivariate normal distribution with zero mean and covariance matrix G, i.e., N(0,G). By incorporating both fixed and random effects, NLME accounts for both inter-individual and intra-individual variability in the data. In pharmacokinetics (PK), Nonlinear Mixed Effects (NLME) models [3] are widely regarded as the state-of-the-art for analyzing and predicting drug concentrations while accounting for individual variability.

Choosing the most suitable model for a given dataset is not always straightforward, and the strong assumptions and approximations underlying these models can restrict their predictive capabilities, potentially limiting their effectiveness in real-world applications. Furthermore, a major limitation of these models is their difficulty in properly incorporating covariates, in particular when dealing with high-dimensional data and complex correlation structures, while selecting the most relevant covariates for the pharmacokinetics of a drug remains a challenge [1].

This study explores an alternative approach to modeling drug concentration curves using Neural Ordinary Differential Equations (NODEs) [5]. Unlike traditional population pharmacokinetic models, NODEs leverage neural networks to learn differential equations directly from data, without relying on predefined assumptions about the system. Another key advantage of this approach is the possibility to directly integrate covariates, allowing the model to capture even complex correlation structures that may be difficult to handle with standard methods. A key advantage of NODEs over other deep learning (DL) techniques becomes particularly evident when predicting drug concentrations for unseen dosing regimens not present in the dataset, a condition for which other DL models often fail [6]. In Bräm et al. (2024) [7], a low-dimensional NODE was integrated directly into popular pharmacometric software such as Monolix 2023R1 and NONMEM 7.5, serving as a support model for the NLME fit within these platforms. This approach was tested on demo datasets, and its robustness on a real-world sparse dataset was not thoroughly evaluated. In fact, unlike conventional deep learning techniques, where models are trained on large datasets to effectively optimize parameter spaces and avoid overfitting, pharmacokinetic studies often involve relatively small sample sizes due to the cost and difficulty of data collection. The limited data size is one of the main challenges in applying NODEs to real-world pharmacokinetic analyses; to address this issue, we propose a data augmentation strategy to pre-train the NODE model, improving its predictions’ stability and generalization. Furthermore, The NODE trained in this work has been designed to capture complex relationships between drug concentration and patient-specific factors, paving the way for more flexible and data-driven precision medicine approaches. The model performances have been compared to the state-of-the-art models, which include compartmental models and NLME.

## 2. Materials and Methods

### 2.1. Materials

The aim of this study was to compare the predictive performances of three approaches for predicting drug pharmacokinetics over time of the antibiotic Dalbavancin, namely, compartment analysis, NLME and NODEs.

The dataset used in this study was collected from a cohort of 218 patients undergoing Dalbavancin treatment and who underwent therapeutic drug monitoring (TDM) of Dalbavancin at the IRCCS, Azienda Ospedaliero Universitaria di Bologna between April 2021 and December 2024. Dalbavancin is a long-acting antibiotic used for the treatment of skin and soft tissue infections and as a second-line agent in patients with staphylococcal infections, such as bone and joint infections, vascular prosthetic joint infections and endocarditis. At our center, Dalbavancin plasma exposure is optimized by means of TDM, which is the medical practice of measuring drug concentration in plasma in order to guide drug dosing for attaining a pre-defined target of efficacy. Dalbavancin is a semisynthetic lipoglycopeptide derived from the teicoplanin-like antibiotic A-40926, which is an agent naturally produced by the actinomycete Nonomuria spp. An important step in the synthesis of Dalbavancin is the amidation of the peptide carboxyl group in the teicoplanin structure with a 3,3 dimethylaminopropylamide group, resulting in the improved potency of Dalbavancin against staphylococci. Moreover, the removal of the acetylglucosamine group of Dalbavancin with respect to teicoplanin resulted in the improved activity against enterococci, even if Van-A enterococci remained resistant to Dalbavancin. The long lipophilic side chain in the Dalbavancin molecule serves a dual purpose by both extending the half-life, as well as anchoring the compound to the membrane, which enhances its antimicrobial activity [8].

Targeting a Dalbavancin plasma concentration above an efficacy threshold, previously defined to be of 8.04mg/L, may be challenged by pharmacokinetic variability. A Bayesian estimation approach aimed at forecasting the duration of concentration persisting over 8.04mg/L was recently developed by means of standard population pharmacokinetics methods (NLME) [9] and validated in a small cohort of patients.

The data acquisition process involved recording patient covariates, drug administration details and serial blood sampling for pharmacokinetic analysis. Dalbavancin doses ranged from 350mg to 1500mg, with a total of 703 administrations recorded. Blood samples were obtained at various time points following drug administration, resulting in 669 concentration measurements. Standardized laboratory procedures were followed for quantifying Dalbavancin levels in plasma, ensuring accuracy and consistency across all samples.

All patients included in the study were adults, with 145 males and 73 females. All the dataset’s covariates collected were: age, height, weight, sex and serum creatine concentration. The values of these covariates are summarized in Table 1.

To ensure robust model evaluation, we employed a cross-validation approach. Given the dataset size and the need for a balance between the training and validation data, we opted for a 6-fold cross-validation framework. This choice provides a good trade-off between statistical reliability and computational efficiency, allowing each model to be trained and validated on multiple subsets of the data while maintaining a sufficient amount of training data per fold.

### 2.2. Methods

The proposed model for fitting drug concentration curves is constructed using Neural Ordinary Differential Equations (NODEs) [5,10].

NODEs are a machine learning technique that extends traditional neural networks by formulating the transformation of hidden states of the neural network (NN) as a continuous-time dynamical system. The right-hand side of a differential equation is substituted with an NN, and then it is integrated through a traditional ODE solver (i.e., Runge–Kutta methods).

To model drug concentration over time, a nonlinear regression approach has been employed. The optimization is performed using the standard squared loss, given by:

(1) $$L=\frac{1}{MN}\sum_{k=1}^{M}\sum_{i=1}^{N_{k}}{\left(y_{k}\left(t_{i}\right)-{\hat{y}}_{k}\left(t_{i}\right)\right)}^{2}$$

where *M* denotes the total number of patients, $N_{k}$ represents the number of concentration measurements for patient *k*, $y_{k}\left(t_{i}\right)$ corresponds to the observed drug concentration at time $t_{i}$ and ${\hat{y}}_{k}\left(t_{i}\right)$ is the predicted concentration obtained from the NODE model. The PyTorch V2.6.0 implementation of differentiable ODE solvers (torchdiffeq [11]) has been used to perform gradient descent and optimize the NN parameter.

Since the compartment models are systems of differential equations, the replacement of these equations with an NN is direct:

(2a) $$\begin{array}{cc} \; & \left\{\begin{array}{c} \frac{dC_{c}}{dt}=-C_{c}\frac{Cl}{V_{1}}-C_{c}\frac{Q}{V_{1}}+C_{p}\frac{Q}{V_{2}}\\ \frac{dC_{p}}{dt}=C_{c}\frac{Q}{V_{1}}-C_{p}\frac{Q}{V_{2}} \end{array}\right. \end{array}$$

(2b) $$\begin{array}{cc} \; & \;\;\;\;\frac{dh(t)}{dt}=f_{nn}\left(h\left(t\right),t;\theta \right) \end{array}$$

The differential equation systems (2a) represent a simple two-compartment model with the four parameters that need to be fitted: $V_{1}$ and $V_{2}$ are the volumes of the central and peripheral compartments, Cl the clearance, *Q* the intercompartmental clearance and $C_{c}$ and $C_{p}$ the concentrations of the two compartments. Equation (2b) shows instead the substitution of all the right-hand side equations with the NN, which, in this case, became a one-dimensional expression; h(t) represents the hidden state of the NODE that, in this work, represents the drug concentration, and θ is the vector of the parameters of the NN.

The specific structure of NNs allows for the direct inclusion of patients’ covariates as model input. In this framework, during training, the NN learns how to integrate covariates into the prediction of concentration curves, inherently capturing all possible correlations. This approach is significantly more efficient than the traditional method of incorporating covariates into compartmental models and NLME. Moreover, it enables a fully data-driven approach, eliminating the need for prior assumptions about the system’s underlying relationships.

The NN represented as $f_{nn}$ in Equation (2b) is a feedforward neural network (FNN), for which two different architectures have been considered in this work. The first and simpler architecture takes four input: time, concentration, number of previous administrations and dose. It produces a single output, i.e., the derivative of the concentration curve. The network includes two hidden layers with 20 neurons each, selected via elbow rule. Different numbers of hidden layers were tested, and two was the smallest number at which the model’s performance reached a plateau; see Appendix A. The activation function used is softplus. This architecture, without covariates, was employed to first compare NODEs with NLMEs and the traditional two-compartment model when provided with the same information. The covariates were not included in the NLME and two-compartment models because, when tested, their inclusion did not improve the results; see Appendix B. Therefore, no covariates have been included in these models. In this way, the comparison between the NODE (both with and without covariates) and the other models was always conducted using the most accurate and well-optimized versions of each model.

The second architecture follows a similar FNN structure but takes nine input variables: the original four together with five covariates. Additionally, an extra hidden layer with 20 neurons was added to take into account the increasing complexity of the model.

To account for multiple administrations, the integration of the NODE is performed piece-wise over the interval between two consecutive doses. The initial condition for each subsequent administration incorporates the residual concentration at that time point, ensuring continuity in the system’s state evolution. This approach effectively treats the problem as a sequence of Initial Value Problems (IVP), each solved independently over its respective subinterval while maintaining consistency across transitions. By adopting this framework, the model captures the cumulative effects of repeated dosing, addressing nonlinearity in drug accumulation and clearance dynamics. The piece-wise integration scheme also facilitates efficient numerical implementation and execution time both for the forward and backward pass. In this way, it is not necessary to integrate the NODE in each administration for a single patient up to the required time to then apply the superposition principle. This results in a more linear and stable gradient computation.

In addition to the main FNN, the model includes an additional parameter, the distribution volume ($V_{NODE}$), which is learned through gradient descent with the whole FNN. This parameter is essential for converting the administered dose into a concentration, analogous to the compartment volume $V_{1}$ in a two-compartment model. However, unlike in the compartmental approach, where two distinct compartments correspond to two separate volumes, the neural ordinary differential equation (NODE) framework employs a single global volume, making direct comparisons between the models unsuitable.

In the case where covariates are included, the distribution volume is estimated using a simple FNN constituted by five input nodes corresponding to the five covariates, a single output and one hidden layer with 10 neurons with a softplus activation function.

**Figure 1:**
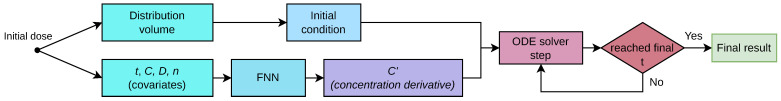
Sketch of the integration process. *t* represents time, *C* the concentration, *D* the dose, *n* the number of previous administrations and final t means the time of the last concentration measure (during training) or the total time for the simulation during inference.

A schematic view of the working principle of the algorithm is summarized in Figure 1.

The dataset employed in this study is characterized by a sparse and irregular sampling of drug concentration measurements across patients. Frequently, patients undergo multiple drug administrations before any pharmacokinetic measurement is recorded, with some measurements occurring only after several doses. This discontinuity and sparsity introduce significant challenges for model training, particularly in maintaining numerical stability and robustness.

To mitigate these issues, a data augmentation strategy was adopted. During each fold of the cross-validation process, a two-compartment pharmacokinetic model was fitted to the available data using nonlinear least squares estimation. The fitted parameters were then used to simulate drug concentration-time profiles under various dosing regimens. From each simulated profile, 50 time points were sampled on a logarithmic time scale to generate synthetic training data. While the use of synthetic data for machine learning is known to down-perform in prediction accuracy and precision with respect to real data trainings [12], in this study, an ablation analysis comparing training with and without synthetic pre-training was attempted. However, training without synthetic data proved largely unstable in this study due to the discontinuous and sparse nature of the original dataset. In most cases, gradient divergence prevented full convergence of the models, making a fair performance comparison unfeasible. Nevertheless, the results suggest that biases introduced by the parametric model during pre-training are effectively mitigated during subsequent fine-tuning on real data, which does not carry the same parametric model biases.

The synthetic data were used to perform an initial pre-training phase for the NODE, aimed at guiding the model to approximate the expected trend of the concentration curve. Following this step, the model underwent fine-tuning using real-world data. The pre-training phase was carried out over 100 epochs using the Adam optimizer [13], with a learning rate of $lr=1\times 10^{-3}$ and a weight decay of $wd=1\times 10^{-5}$. The fine-tuning process began with an additional 100 epochs using the same optimizer and hyper-parameter settings. Subsequently, the model was trained for another 100 epochs, with a reduced learning rate of $lr=1\times 10^{-4}$.

The performance of the resulting NODE has been compared to two of the most widely used models in pharmacokinetics: the compartment models and the NLME models.

**Figure 2:**
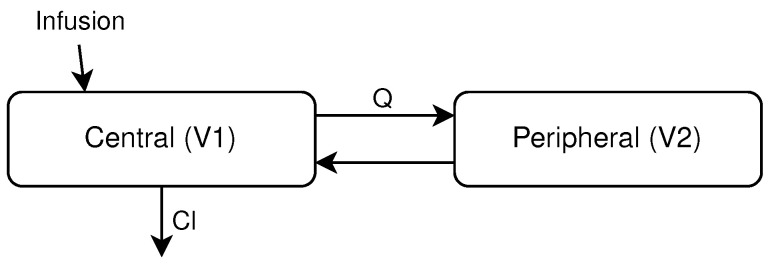
Scheme of a two-compartment model. The antibiotic is administered in the central compartment, which is the only compartment that can eliminate the antibiotic at rate Cl, the clearance. Q is the intercompartmental clearance, while V1 and V2 are the compartment volumes.

In the case of Dalbavancin, multiple compartment models with varying numbers of compartments were evaluated, and the two-compartment model, outlined in Figure 2, has been chosen for the comparison since it provided the best performances [14,15]. For the two-compartment model, the following parameters need to be estimated: the volumes of the two compartments (V1 and V2), the intercompartmental clearance (*Q*) and the clearance (Cl).

In this work, the fitting procedures of the two compartment models have been performed via nonlinear least squares (NLLS).

For what concerns the NLME model, the Monolix software [16] has been used to perform the fitting and compute the predictions. The popPK model settings used in Monolix are: two-compartment distribution, infusion, no delay and linear elimination, where the drug is eliminated from the central compartment at a rate proportional to its concentration. This is represented by a linear dependence on the concentration of the central compartment in the ODE that represents the system (Equation (2a)). Covariates can also be incorporated into the model for each parameter. Typically, when a covariate is included in the model through a specific parameter (let $\theta_{i}$ be that parameter), an additional population parameter, $\alpha_{\theta_{i}}$, is introduced. This parameter is modeled exponentially as $e^{\alpha_{\theta_{i}}}$ and is estimated along with the other parameters. To estimate the model parameters, Maximum Likelihood Estimation has been used.

Finally, in many fields, particularly in clinical applications, model explainability can be just as crucial as prediction accuracy. Over the years, several methods have been developed to improve explainability. Many of these algorithms rely on similar underlying assumptions and can be integrated into a unified framework: Shapley Additive Explanations (SHAP) [17]. This framework aims to quantify the contribution of each input feature through the computation of Shapley values [18]. In this work, these values were calculated for both NODE configurations, with and without covariates. The Shapley values provide insights into the model’s behavior and enable an analysis of the models personalization capabilities.

## 3. Results

**Figure 3:**
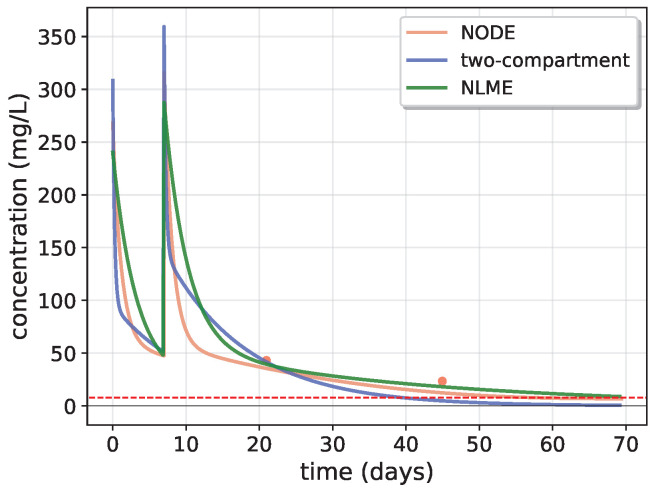
Comparison of the concentration prediction of NODE with respect to the two-compartment model and NLME for a test patient (for a fixed cross-validation iteration). The red dashed line represents the drug efficacy threshold, fixed to t=8.04mg/L, while red dots are real data, corresponding to drug concentration measurements, and the spikes correspond to dose administrations.

To ensure a robust comparison and validation of NODE predictions against NLME and the two-compartment model, a 6-fold cross-validation approach was employed. This comparison has been performed through residual analysis of the three different models. As already stated in Section 2.2, the two-compartment model and the NLME model have been trained without covariates because they guaranteed the most robust and most consistent performances with respect to the same models trained, including covariates (see Appendix B). In Figure 3, the comparison of the three models in predicting the Dalbavancin concentration in time is shown for a patient, as an example, evaluated in the specific iteration of the cross-validation, in which the patient is assigned to the test fold, highlighting the differences in predictions, especially for short and long time ranges. The Dalbavancin efficacy threshold (represented by the red dashed horizontal line in the figure) is set to t=8.04mg/L, according to Cojutti et al. (2024) [9,14,15].

Residuals were computed for model evaluation and comparison. Given the substantial variation in concentration values between short and long time ranges, model performance was assessed using both absolute residuals:

(3) $$r=C_{data}-C_{pred}$$

and relative residuals:

(4) $$r_{rel}=\frac{C_{data}-C_{pred}}{C_{data}}$$

where $C_{data}$ represents the measured concentration and $C_{pred}$ denotes the predicted value.

**Figure 4:**
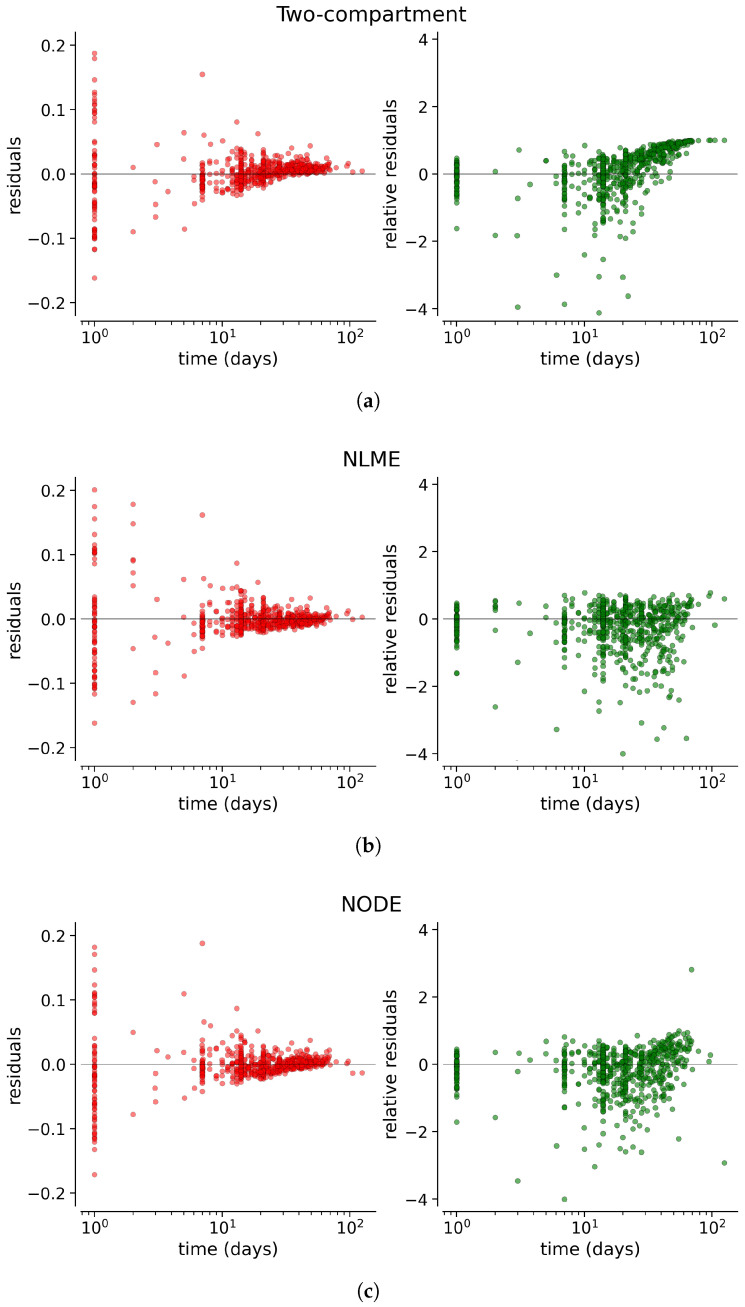
Absolute residuals (**left**) and relative residuals (**right**) as a function of time for the two-compartment model (**a**), the NLME model (**b**) and the NODE (**c**). Each administration resets the time.

Figure 4 shows the absolute and relative residuals of the three models as a function of time, highlighting a bias in long time predictions for the two-compartment model. To enhance visualization, time is reset to zero at each administration, so that the *x*-axis will represent the time since the most recent dose. All residuals have been computed for patients belonging to the test fold from each cross-validation iteration.

**Figure 5:**
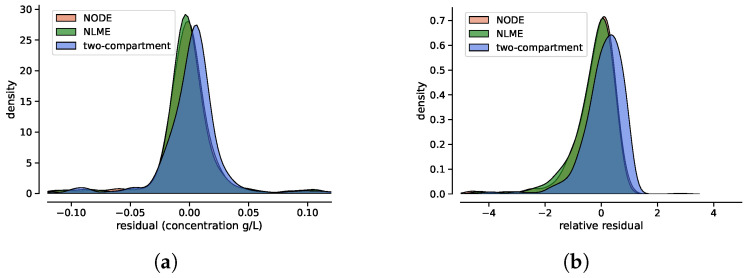
Distributions (via kernel density estimation) of the absolute residuals (**a**) and the relative residuals (**b**) for the two-compartment model, the NLME and the NODE.

Figure 5 presents the kernel density estimations of the residual and relative residual distributions for the three techniques. To assess whether these distributions differ, a two-sample Kolmogorov–Smirnov (KS) test [19] was performed, with the results reported in Table 2.

**Figure 6:**
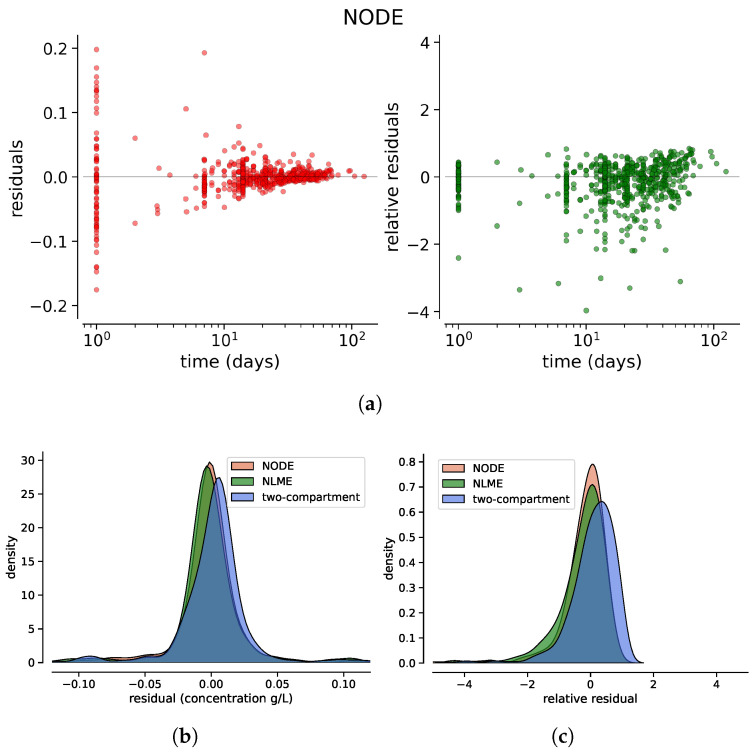
Residuals as a function of time (**a**) and the distributions (via kernel density estimation) of the absolute residuals (**b**) and relative residuals (**c**) for the two-compartment model, the NLME and the NODE trained, including the patients’ covariates.

As already stated in Section 2.2, the NODE model has been trained to also include the covariates of the patients. Figure 6 shows the scatter plot of the residuals and the kernel density estimations of the residual and relative residual distributions for this model, compared to the two-compartment and NLME models, which were trained without covariates since this setting yields the best performances for those models (Appendix B).

To assess whether these distributions differ, a KS test was performed, with the results reported in Table 3.

The *p*-value for NLME and the two-compartment model are not reported in Table 3 since it is equal to the one reported in Table 2.

Overfitting was evaluated and found to be not significant, as demonstrated in Appendix C, where the results are reported.

The estimated parameters for the two-compartment model and the NLME are shown in Table 4 and compared with the literature for *Dalbavancin* [15].

On the other hand, the FNNs used for modeling the NODE do not yield parameters directly comparable to those reported in Table 4. In the NODE model without covariates, the *distribution volume* $V_{NODE}$ is treated as a population parameter, as described in Section 2.2, and can be compared to the central compartment volume $V_{1}$ in a two-compartmental model. The estimated value of $V_{NODE}$ is:

(5) $$V_{NODE}=5.7\pm 0.1\;L$$

When covariates are included, however, $V_{NODE}$ becomes an individual-specific parameter, estimated by the FNN for each patient based on their covariate profile. It is therefore no longer a fixed population parameter and can vary during inference depending on the input. As a result, it is not directly comparable to the population parameters previously discussed. Nonetheless, despite exhibiting greater variability, the values estimated for $V_{NODE,\;\mathrm{cov}}$ remain consistent with physiologically plausible distribution volumes. Specifically, for the patients in the test set, its mean and standard deviation are:

(6) $$V_{NODE,\;\mathrm{cov}}=5.2\pm 0.8\;L$$

Finally, model explainability was addressed using SHAP values, which were computed for both NODE models, trained with and without the patients’ covariates. SHAP values were computed separately for each iteration of the cross-validation for the test folder. However, these values were concatenated across all iterations and presented in a single plot. The trends observed in the plot remain consistent across the different iterations. It is important to note that SHAP values were computed with respect to the FNN component of the NODE model, which represents the right-hand side of the ODE, that is, the derivative of the concentration curve. Therefore, the SHAP values reflect the contribution of each input feature to the predicted rate of change in drug concentration.

**Figure 7:**
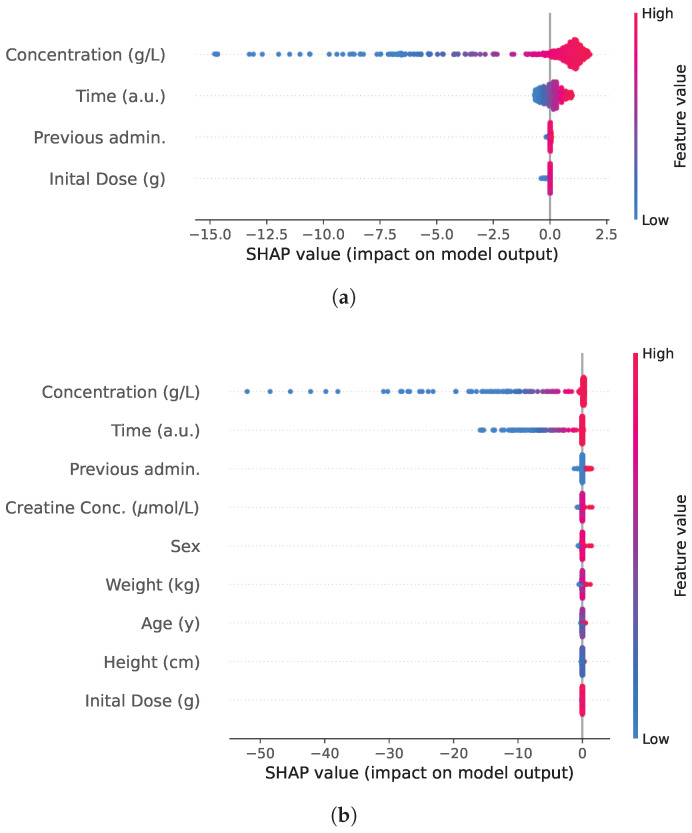
(**a**) Beeswarm plot of SHAP values for each input feature in the NODE model without covariates. (**b**) Beeswarm plot of input features in the model with covariates, indicating their minimal impact on the output. The color maps give an insight on the relation between SHAP values and feature values.

These SHAP values are shown in Figure 7.

**Figure 8:**
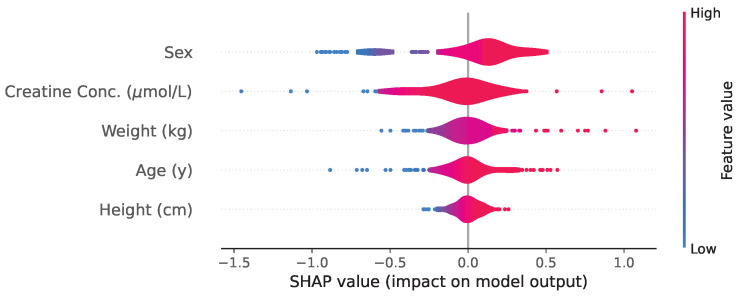
Violin plot of the SHAP values used in the computation of $V_{\mathrm{NODE}}$ for the model with covariates, where volume is treated as an individual-specific parameter, illustrating a comparable contribution from each covariate.

For the NODE trained with covariates, the distribution volume $V_{NODE,\;\mathrm{cov}}$ is also estimated based on covariates, meaning it is no longer a population parameter. Thus, its values vary during inference, and SHAP values can be computed to assess the impact of each different covariate in its estimation, as illustrated in Figure 8.

## 4. Discussion

This study explores the effectiveness of NODE in modeling drug pharmacokinetics compared to the traditional two-compartment model and NLME, the state-of-the-art method in pharmacokinetics. The results indicate that NODE offers a promising alternative, yielding comparable performances to NLME when trained without patients’ covariates, while it outperforms both NLME and the two-compartment model when trained with these covariates.

The analysis of residuals highlights key differences among the three modeling approaches. The two-compartment model systematically underestimates long-term drug concentrations, as evidenced by the obvious trend in relative residuals (Figure 4). In contrast, NODE and NLME exhibit highly similar residual distributions, as confirmed by their overlapping KDE distributions and the KS test (p=0.51), which indicates no statistically significant difference. However, the test results confirm that the residuals of the two-compartment model follow a distinctly different distribution. Moreover, these results indicate that although the NODE initially learns a biased trend during pre-training with the simple two-compartment model, fine-tuning with real data effectively corrects this behavior and eliminates this bias.

Without covariates, NODE does not provide an improvement in predicting drug concentrations with respect to state-of-the-art NLME. While NODE could, in principle, offer a more flexible approximation of the underlying ODE system compared to compartmental models, no such improvement was observed in this scenario. When covariates are included, NODE shows a enhanced performance, as reflected in the relative residuals, where the KDE distribution exhibits a higher peak, suggesting reduced variability and a better fit to the data. The KS test (p=0.008) further confirms a statistically significant difference in residual distributions when covariates are included.

For what concerns the parameter estimates (Table 4), while the two-compartment model fails to accurately estimate the inter-compartmental clearance *Q*, the NLME produces results consistent with previous literature estimations [15]. Additionally, the distribution volumes $V_{NODE}$ (Equation (5)) and $V_{NODE,\;\mathrm{cov}}$, although not directly comparable to $V_{1}$, the volume of the first (central) compartment, assumes similar values. This outcome aligns with expectations, as $V_{NODE}$ serves a similar role to $V_{1}$, facilitating the conversion of administered doses into concentrations and vice versa.

The SHAP value analysis provides insights into the explainability of the NODE model. For the NODE trained without covariates, concentration and time emerge as the most influential input, as expected, while the number of previous administrations and the initial dose have minimal impact. When covariates are included, concentration and time remain the primary determinants of the predicted concentration curves, while other input, including patients’ covariates, have a limited influence. The slightly sharper peak in the KDE distribution of residuals when covariates are included, combined with the SHAP value analysis, suggests that covariates primarily function as correction factors rather than key predictive variables.

Hence, while NODE demonstrates performance comparable to that of NLME, its advantage remains limited in the absence of covariates. The inclusion of covariates enhances prediction accuracy, and their influence acts as a corrective adjustment rather than a fundamental shift in the model’s behavior.

An important implication of our findings is the potential for Neural ODEs to serve as a robust tool in settings where data sparsity and complex covariate interactions limit the applicability of traditional NLME models. The superior performance of NODEs with covariate integration highlights their ability to capture nonlinear and potentially non-additive relationships that are often oversimplified in parametric approaches. Moreover, the use of SHAP values contributes to improving model interpretability, addressing a common criticism of deep learning applications in clinical contexts. This not only enhances the transparency of predictions, but also facilitates clinical trust and regulatory acceptance.

## 5. Conclusions

This study demonstrates that NODE models offer a promising alternative to traditional methods, the compartmental and NLME models, for analyzing drug pharmacokinetics.

The incorporation of covariates into the NODE models’ input led to enhanced predictive performance, as proved by the statistically significant KS test result used to compare the residual distribution of the NODE predictions with respect to the other models. These improvements suggest that NODE effectively leverages covariates as correction factors, leading to more accurate concentration estimations and reduced variability. Furthermore, the explainability of the model was addressed via SHAP values, confirming that its behavior aligns with expectations and remains interpretable.

In conclusion, NODEs represent a viable and complementary alternative to NLMEs in pharmacokinetics, offering flexibility in modeling complex relationships while maintaining explainability. Future research should explore NODEs’ potential to handle more complex pharmacokinetic scenarios and assess their scalability on larger datasets. Furthermore, investigating advanced NODE-based models, such as Neural Stochastic Differential Equations (NSDEs) [10] and Neural Controlled Differential Equations (NCDEs) [20], could further extend their applicability and robustness in pharmacokinetic modeling.

## Figures and Tables

**Table 1:** Summary description of the Dalbavancin dataset.

Covariate	Range	Mean ± Std
Age (y)	[18,92]	64±16
Height (cm)	[145,190]	171±9
Weight (kg)	[40,140]	77±16
Creatine conc. (μmol/L)	[19,411]	94±48

**Table 2:** Kolmogorov–Smirnov test *p*-values for the relative residual distributions. The *p*-values were adjusted for multiple comparisons using the Benjamini–Hochberg procedure.

KS-Test Comparison	*p*-Value
NODE and NLME	0.51
NODE and two-comp.	$2.5\times 10^{-18}$
NLME and two-comp.	$1.4\times 10^{-19}$

**Table 3:** Kolmogorov–Smirnov test *p*-values for the relative residual distributions of the NODE trained with covariates. The *p*-values were adjusted for multiple comparisons using the Benjamini–Hochberg procedure.

KS Test Comparison	*p*-Values
NODE (with covariates) and NLME	0.008
NODE (with covariates) and two-comp.	$2.2\times 10^{-17}$

**Table 4:** Mean values of the estimated parameters for the two-compartment model and NLME among the six iterations of the cross-validation. The associated error is the standard deviation.

Parameter	Two-Compartment	NLME Model	Literature [15]
Cl (L/h)	0.054±0.002	0.0367±0.0006	0.031±0.004
*Q* (L/h)	0.42±0.14	0.028±0.003	0.038±0.013
$V_{1}$ (L)	5.3±0.4	6.32±0.15	5.9±0.3
$V_{2}$ (L)	8.8±1.5	13.9±0.6	9.6±1.4

**Figure A1:**
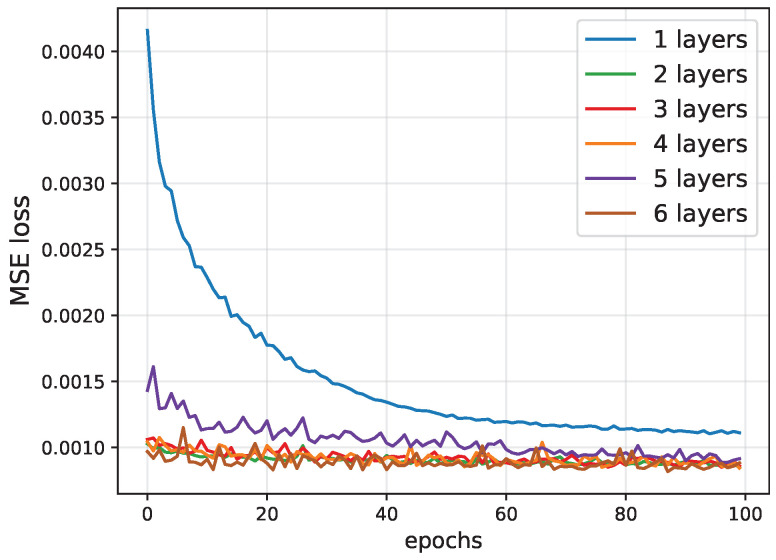
Loss function profiles for training with different number of parameters.

**Figure A2:**
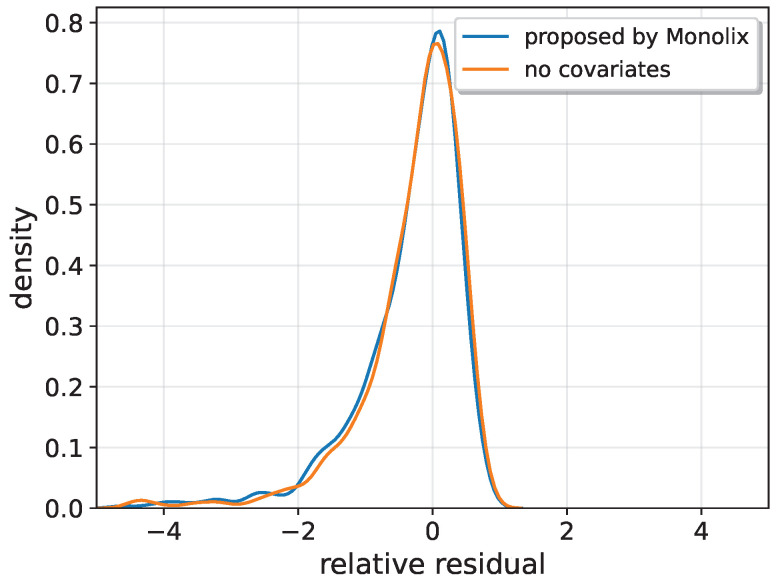
Comparison of residuals of the best model fitted in Monolix with the one without covariates.

**Figure A3:**
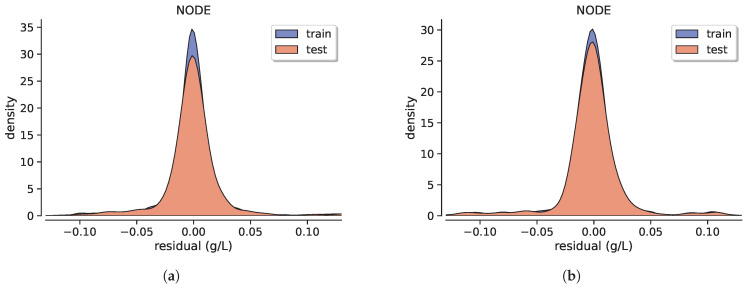
Comparison of distributions from a kernel density estimations of the residuals of the NLME (**a**) and the residuals of the NODE (**b**).

## Data Availability

The data presented in this study are available on request from the corresponding author due to privacy reasons. The python 3.10 implementation code of the algorithm described in the paper can be found on GitHub: https://github.com/TommyGiak/pharmacoNODE (accessed on 6 May 2025).

## Abbreviations

The following abbreviations are used in this manuscript:

NODE	Neural Ordinary Differential Equations
NLME	Nonlinear Mixed Effects
NN	Neural Network
FNN	Feedforward Neural Network
DL	Deep Learning
KDE	Kernel Denisity Estimation
KS	Kolmogorov–Smirnov
SHAP	Shapley Additive Explanations

## Appendix A. Elbow Method

The optimal number of layers and neurons was determined using the elbow method. A preliminary training was conducted with varying numbers of layers, and the model with the fewest parameters that achieved the performance plateau was selected (Figure A1). The model reached the plateau with just two layers.

## Appendix B. Covariates’ Inclusion in NLME Model

Comparison of residuals between the NLME model trained in Monolix without covariates and the model trained with the most correlated variables at each iteration of cross-validation (Figure A2).

The KS test for the two distributions yields a *p*-value of 0.45, indicating no significant difference between them. Consequently, while we acknowledge that the exclusion of clinically relevant covariates may limit the physiological interpretability of the NLME model, we preferred to select the less complex model since it represents a robust and scalable version of the model and does not rely on the specific choices of the covariates to include for each parameter, which vary a lot depending on the specific iterations of the cross-validation.

## Appendix C. Overfitting

To evaluate overfitting for the NODEs and the NLME models the residuals computed on the test set were compared to the ones computed on the train set. Figure A3 shows this comparison through the densities from the kernel density estimation.

The KS-test performed between the NLME and the NODE without covariates gives a *p*-value of 0.92 while the test performed between NLME and the NODE with covariates yields a *p*-value of 0.74.

To further validate the absence of overfitting, the mean squared error (MSE) and $R^{2}$ score were computed for both test and train data and then compared, Table A1 and Table A2.

**Table A1:** MSE computed for comparing the model performances in test and train set for both settings with and without covariates.

Model	Test MSE	Train MSE
without covariates	0.001±0.004	0.001±0.004
with covariates	0.001±0.005	0.001±0.004

**Table A2:** $R^{2}$ score computed for comparing the model performances in test and train set for both settings with and without covariates.

Model	Test $R^{2}$	Train $R^{2}$
without covariates	0.850	0.854
with covariates	0.830	0.870
